# Improved calibration estimators for the total cost of health programs and application to immunization in Brazil

**DOI:** 10.1371/journal.pone.0212401

**Published:** 2019-03-06

**Authors:** Claudia Rivera-Rodriguez, Cristiana Toscano, Stephen Resch

**Affiliations:** 1 Department of Statistics, The University of Auckland, Auckland, New Zealand; 2 Department of Community Health, Institute of Tropical Pathology and Public Health, Federal University of Goiás (UFG), Goiás, Brazil; 3 Center for Health Decision Science, Harvard T.H. Chan School of Public Health, Boston, MA, United States of America; US Department of State Bureau of Diplomatic Security, UNITED STATES

## Abstract

Multi-stage/level sampling designs have been widely used by survey statisticians as a means of obtaining reliable and efficient estimates at a reasonable implementation cost. This method has been particularly useful in National country-wide surveys to assess the costs of delivering public health programs, which are generally originated in different levels of service management and delivery. Unbiased and efficient estimates of costs are essential to adequately allocate resources and inform policy and planning. In recent years, the global health community has become increasingly interested in estimating the costs of immunization programs. In such programs, part of the cost correspond to vaccines and it is in most countries procured at the central level, while the rest of the costs are incurred in states, municipalities and health facilities, respectively. As such, total program cost is a result of adding these costs, and its variance should account for the relation between the totals at the different levels. An additional challenge is the missing information at the various levels. A variety of methods have been developed to compensate for this missing data. Weighting adjustments are often used to make the estimates consistent with readily-available information. For estimation of total program costs this implies adjusting the estimates at each level to comply with the characteristics of the country. In 2014, A National study to estimate the costs of the Brazilian National Immunization Program was initiated, requested by the Ministry of Health and with the support of international partners. We formulate a quick and useful way to compute the variance and deal with missing values at the various levels. Our approach involves calibrating the weights at each level using additional readily-available information such as the total number of doses administered. Taking the Brazilian immunization costing study as an example, this approach results in substantial gains in both efficiency and precision of the cost estimate.

## Introduction

The global health community has become increasingly interested in estimating the costs of delivering public health programs, which are generally originated in different levels of service management and delivery. The interest has arisen from the need for financial sustainability and expansion of these programs. However, in low and middle income countries the resource utilization and financial information is scarce. Among the reasons for the lack of such information is the fact that public health program implementation occurs mostly at decentralized levels of management, and many costs are shared with other programs. As a result, program managers do not have the key information to improve efficiency or to advocate for adequate budget support. This is even more important when considering newly available technologies that are incorporated into public health programs, such as the introduction of new vaccines which are significantly more costly that the traditional childhood vaccines [1–4].

The costs of immunization programs occurs in various levels, where part of the cost correspond to vaccines and it is in most countries procured at the central level, while the rest of the costs are incurred in states, municipalities and health facilities, respectively. As such, total program cost is a result of adding these costs, and its variance should not only account for the uncertainty at each level, but also for the uncertainty due to the totals at each level being related (or nested) and for missing data.

In the past several years, selected studies have been conducted to assess the costs of immunization programs. In particular, various low and middle income countries conducted immunization costing studies since 2010, with the support from the Expanded Program on Immunization Costing and Financing (EPIC) project [1]. Detailed costing information on routine child immunization from Benin, Ghana, Honduras, Moldova, Uganda and Zambia [2, 3, 5–8]. These studies had in common the fact that they aimed at estimating total immunization costs, and not only expenditures on immunization. They were characterized by using multi-stage sampling of facilities and regression modelling to estimate the average costs at the various levels. The estimated average cost was then multiplied by the nationwide number of corresponding units (e.g. districts, facilities, etc.) to estimate total program costs. The study conducted in Honduras used sampling weights to obtain estimates of total national costs for routine immunization [3]. Despite having many strengths, the methods require that information is not missing, and does not allow generating a measure of uncertainty for the estimated total immunization program cost.

Over recent years, a variety of methods have been developed to compensate for missing data [9–17]. Weighting adjustments are often used to make the estimates consistent with readily-available information. For estimation of total program costs this implies adjusting the estimates at each level to comply with the characteristics of the country. Due to the need for collecting data at various levels, the large number of health-care units providing immunization at the local level, and considering the wide diversity of cost categories in immunization programs, it is inevitable that selected data will be lacking when conducting a comprehensive costing survey of immunization programs. Such missing data may be from the sampling units at any of the levels surveyed. This, coupled with the lack of an uncertainty measure of the overall cost estimate, represents the major methodological challenges when conducting nationwide immunization costing studies. Obtaining a precise and accurate cost estimator is critical for decision makers to inform policy and planning, and to recommend future studies.

Brazil structured its National Immunization Program in the 70’s, and has since strengthened the program with legislation to secure funds. Various newly available vaccines have been incorporated into the program in the past decade, taking into consideration recommended framework for vaccine introduction decision-making [4]. In order to generate evidence to support decision making and strengthen national capacity to appropriately inform policy and planning on the introduction of new vaccines, the Brazilian ministry of health commissioned a study to assess the costs of the Brazilian National immunization Program. A nationwide costing survey was conducted considering the year of 2013 and the perspective of SUS, the Brazilian National Public Health-care system which provides immunization services free of charge to all population through its more than 35,000 health-care services distributed in the country’s 5 macro-regions and 27 states (including a federal district) [4]. More details of the methods can be found in [6].

In brief, data on all resources used in the immunization activities which occur at four organizational levels was collected: central, state, municipal, and health care facilities providing immunization services. Data was collected at the central and all 27 state levels, in addition to a sample of municipalities and health-care facilities. The sample was collected following a two-stage stratified scheme, where the strata and stages are based on regions and municipalities respectively. After data collection, selected data was missing at both the facility and municipality levels.

Using the Brazilian National Immunization Program Costing Study, we propose and validate a quick and useful approach to estimating the inter-level variance while accounting for missing data at different sampling levels. Our method involves calibrating the weights at each level using additional readily-available information for the corresponding entire level such as the total number of doses administered or population targeted by the immunization program. Furthermore, we present theoretical calculations on how to compute the variance of this estimator using Taylor approximations. The paper is composed as follows. The first section describes the total cost and its components, the second section describes the sampling design and missing data, followed by estimation methods and variance. This is followed by post-design methods to improve estimation of the total cost and we finalize the paper with application to the Brazilian immunization study.

## The total cost *T*

The main interest lies in estimating the total cost of a health program, e.g. the routine immunization program in Brazil. This cost can be expressed as

$$\begin{array}{c} T=T_{\text{C}}+T_{\text{F}}+T_{\text{M}}+T_{\text{R}}, \end{array}$$ (1)

where *T*_F_, *T*_M_ and *T*_R_ represent the total costs at the facility level, municipality level and regional level, respectively. Note that *T*_F_, *T*_M_, or *T*_R_ are not necessarily unknown, but it is usually the case that at least two of them are to be estimated [2, 3, 5, 7, 8]. *T*_C_ represents the additional cost accrued centrally and it is assumed known. For example, in Brazil, this cost arises from vaccines purchased at the central level, in addition to coordination and management activities incurring in personnel, transportation and infrastructure costs. Note that, if desired, this expression can also be used to estimate any total figure of interest. Usually costing studies can have different goals, but, in order to simplify and facilitate the readability of the paper, we focus on total cost. We denote *N*_R_ as the total number of regions, $N_{M_{r}}$ as the total number of municipalities within region *r* and $N_{F_{r}m}$ is the total number of facilities in municipality *m*, region *r*. Each of these costs can, respectively, be expressed as follows:

$$\begin{array}{c} T_{\text{R}}=\sum_{r=1}^{N_{\text{R}}}y_{R_{r}}, \end{array}$$ (2)

where $y_{R_{r}}$ is the total cost specific to regions and *N*_R_ is the number of regions. Similarly, *T*_M_ and *T*_F_ can be represented as

$$\begin{array}{c} T_{\text{M}}=\sum_{r=1}^{N_{\text{R}}}\sum_{m=1}^{N_{{\text{M}}_{r}}}y_{{\text{M}}_{rm}} \end{array}$$ (3)

$$\begin{array}{c} T_{\text{F}}=\sum_{r=1}^{N_{\text{R}}}\sum_{m=1}^{N_{{\text{M}}_{r}}}\sum_{f=1}^{N_{F_{mr}}}y_{{\text{F}}_{rmf}}, \end{array}$$ (4)

where $y_{{\text{M}}_{rm}}$ is the total cost incurred by municipality *m* in region *r* and $y_{{\text{F}}_{rmf}}$ is the total cost specific to facility *f* in municipality *m* in region *r*. Note, this expression explicitly acknowledges that facilities are structured as belonging to some specific municipality within some specific region. Furthermore, the number of municipalities within any given region may vary across regions, and the number of facilities within any given municipality may also vary. Taking this structure into consideration is essential for valid estimation and inference.

## The sampling design and missing data

Generally, the costs incurred at region, municipality and facility levels are unknown. Researchers usually have access to readily available information on the hierarchical structure or demographic information at each level, but not to cost data. It is often the case that investigators have access or resources to collect detailed information on costs in a sub-sample of units. A cost-efficient approach is to implement a multi-stage sampling design where a sample of units is selected at each stage and the costs/variables of interest are recorded for each sampled unit. For example, in Brazil, the first stage units are regions and a sample of *n*_R_ = *N*_R_ regions was selected. Because all regions were included in the sample, this is the same as a stratified sampling design. Subsequently, a sample of municipalities was selected from each region. The sampling design within each region was proportional to the number of children under one year old, with an expected sample size of $E(n_{{\text{M}}_{r}})$, where $n_{{\text{M}}_{r}}$ denotes the final number of municipalities sampled. Additionally, within each municipality, a random sample of $n_{{\text{F}}_{rm}}$ facilities was selected. The expected municipality sample size per macro-region was 11 (55 in total, 5 macro regions), and the estimated facility sample size was 66 immunization services per macro-region, totaling 330 immunization services in the whole country. At each macro region, the facilities from the 11 selected municipalities were assembled into a pool. Then 66 facilities were selected from each pool. Due to this sampling mechanism, not all municipalities had facilities selected. In fact, this yielded a final sample of 330 vaccination units located in 40 Brazilian municipalities. Municipalities for which no health facilities were selected in the second stage of the sampling process were not visited and therefore did not have the municipal level data collected (15 municipalities). In addition, the information on two municipalities and its facilities was lost due to logistic issues. These municipalities were Inocência, in the central-west region, and Riachão do Jacuṕe in the Northeastern Region. This implies that the information from facilities belonging to these two municipalities was also missing.

Table 1(1) shows the final number of municipalities and facilities per region. For the Brazilian study, the total expected number of sampled municipalities was $E(n_{{\text{M}}_{r}})=55$, but the actual size was $\sum_{r}n_{{\text{M}}_{r}}=38$, while the actual sample size of facilities was $\sum_{r,m}n_{{\text{F}}_{rm}}=325$.

**Table 1:** Final number of municipalities and facilities per region.

Region	No. mun.	No. mun. sampled	No. fac.	No. fac. sampled
Midwest	464	10	1988	64
Northeast	1787	6	10897	64
North	447	9	2103	65
Southeast	1663	7	8312	66
South	1182	6	4087	66

## IPW (Inverse probability weighting) estimation

In absence of complete data at municipality and facility levels, neither *T*_R_, *T*_M_ nor *T*_F_ can be directly calculated. This is the case of multistage sampling design of our interest, where the total costs cannot be calculated. A possible approach for overcoming this limitation allowing for total costs estimation is the well-known inverse probability weighting (IPW) [10, 18]. It uses sampling weights to build a bridge between the observed subsample and the entire population to produce an estimator that represents all the population. Let $n_{F_{mr}}$ the total number of facilities sampled in municipality *m* in region *r* and $n_{{\text{F}}_{r}}$ be the total number of facilities sampled in region *r*. Focusing on *T*_R_, the IPW estimate is given by

$$\begin{array}{c} {\hat{T}}_{\text{R}}=\sum_{r=1}^{N_{\text{R}}}I_{R_{r}}w_{r}y_{R_{r}}, \end{array}$$ (5)

where $I_{R_{r}}$ is an indicator of whether the region *r* was selected and $w_{R_{r}}=1/\pi_{R_{r}}$, with *π*_R_ the probability that region *r* was selected to be part of the survey. In Brazil, for example, $I_{R_{r}}=1$ for all five regions and $w_{R_{r}}=1$. In some settings, such in the Honduras EPIC study, not all regions were included [3]. In such situations, some $I_{R_{r}}$ are zero. Similarly, an IPW estimator of *T*_M_ is

$$\begin{array}{c} {\hat{T}}_{\text{M}}=\sum_{r=1}^{N_{\text{R}}}\sum_{m=1}^{N_{{\text{M}}_{r}}}I_{{\text{M}}_{rm}}w_{{\text{M}}_{rm}}y_{{\text{M}}_{rm}}, \end{array}$$ (6)

where $I_{{\text{M}}_{rm}}$ is an indicator of whether municipality *m* in region *r* was selected at the second stage sample, and $w_{{\text{M}}_{rm}}=1/\pi_{{\text{M}}_{rm}}$ with $\pi_{{\text{M}}_{rm}}$ being the probability that the municipality was selected. In Brazil, 40 municipalities were selected initially, but the information on one of them is missing, then only 38 are available for the analysis.

Similarly, an IPW estimator of *T*_F_ is

$$\begin{array}{c} {\hat{T}}_{\text{F}}=\sum_{r=1}^{N_{\text{R}}}\sum_{m=1}^{N_{{\text{M}}_{r}}}\sum_{f=1}^{N_{F_{mr}}}I_{{\text{F}}_{rmf}}w_{{\text{F}}_{rmf}}y_{{\text{F}}_{rmf}}, \end{array}$$ (7)

where $I_{{\text{F}}_{rmf}}$ is an indicator of whether facility *f* in municipality *m*, in region *r* was selected at the third stage sample, and $w_{{\text{F}}_{rmf}}=1/\pi_{{\text{F}}_{rmf}}$ with $\pi_{{\text{F}}_{rmf}}$ as the inclusion probability for such facility. The final IPW estimator is then given by $\hat{T}=T_{\text{C}}+{\hat{T}}_{\text{F}}+{\hat{T}}_{\text{M}}+{\hat{T}}_{\text{R}}$. Under a true design, statistical properties guarantee $\hat{T}$ to be an unbiased estimator of the total cost *T* [10, 18].

### Variance estimation

Usually IPW estimators only involve data at the last stage of the sampling design. An example of this is ${\hat{T}}_{\text{F}}$. In this case, the variance of the estimator is well known and straight forward to calculate following standard properties of multi stage designs [18]. However, our estimator of interest involves information from the various stages of the sampling design. This implies that the variance has to be estimated differently. Note first that

$$\begin{array}{c} I_{{\text{F}}_{rmf}}=I_{{\text{F}}_{rmf}}I_{{\text{M}}_{rm}}I_{{\text{R}}_{r}};\;I_{{\text{M}}_{rm}}=I_{{\text{M}}_{rm}}I_{{\text{R}}_{r}}. \end{array}$$ (8)

Using this, the number of facilities sampled in municipality *m*, region *r* can be written as $n_{{\text{F}}_{rm}}=\sum_{f=1}^{N_{{\text{F}}_{rm}}}I_{{\text{F}}_{rmf}}$ and the number of facilities sampled in region *r* can be written as $n_{{\text{F}}_{r}}=\sum_{m=1}^{N_{{\text{F}}_{r}}}I_{{\text{M}}_{rm}}\sum_{f=1}^{N_{{\text{F}}_{rm}}}I_{{\text{F}}_{rmf}}I_{{\text{F}}_{rmf}}$ and these quantities are fixed by design. Furthermore, note that ${\hat{T}}_{\text{R}},{\hat{T}}_{\text{M}},{\hat{T}}_{\text{F}}$ (Eqs (5), (6) and (7)) can respectively be written as

$${\hat{T}}_{\text{R}}=\sum_{r=1}^{N_{\text{R}}}I_{R_{r}}\sum_{m=1}^{N_{{\text{M}}_{r}}}I_{{\text{M}}_{rm}}\sum_{f=1}^{N_{F_{mr}}}I_{{\text{F}}_{rmf}}\frac{w_{{\text{F}}_{rmf}}w_{R_{r}}y_{R_{r}}}{w_{{\text{F}}_{rmf}}n_{{\text{F}}_{r}}},$$ (9)

$${\hat{T}}_{\text{M}}=\sum_{r=1}^{N_{\text{R}}}I_{R_{r}}\sum_{m=1}^{N_{{\text{M}}_{r}}}I_{{\text{M}}_{rm}}\sum_{f=1}^{N_{F_{mr}}}I_{{\text{F}}_{rmf}}\frac{w_{{\text{F}}_{rmf}}w_{{\text{M}}_{rm}}y_{{\text{M}}_{rm}}}{w_{{\text{F}}_{rmf}}n_{F_{mr}}},$$ (10)

$${\hat{T}}_{\text{F}}=\sum_{r=1}^{N_{\text{R}}}I_{R_{r}}\sum_{m=1}^{N_{{\text{M}}_{r}}}I_{{\text{M}}_{rm}}\sum_{f=1}^{N_{F_{mr}}}I_{{\text{F}}_{rmf}}w_{{\text{F}}_{rmf}}y_{{\text{F}}_{rmf}}.$$ (11)

Now, let

$$\begin{array}{c} u_{rmf}=y_{{\text{F}}_{rmf}}+\frac{w_{{\text{M}}_{rm}}}{w_{{\text{F}}_{rmf}}n_{F_{mr}}}y_{{\text{M}}_{rm}}+\frac{w_{R_{r}}}{w_{{\text{F}}_{rmf}}n_{{\text{F}}_{r}}}y_{{\text{R}}_{r}}. \end{array}$$ (12)

Then, using (9)–(11) and *u*_*rmf*_, we can write the estimator $\hat{T}$ as

$$\begin{array}{c} \hat{T}=\sum_{r=1}^{N_{\text{R}}}\sum_{m=1}^{N_{{\text{M}}_{r}}}\sum_{f=1}^{N_{F_{mr}}}I_{{\text{F}}_{rmf}}w_{{\text{F}}_{rmf}}u_{rmf}. \end{array}$$ (13)

Since $n_{{\text{F}}_{r}}$, $n_{{\text{F}}_{rm}}$, $w_{{\text{M}}_{rm}}$ and $w_{{\text{F}}_{rmf}}$ are fixed by design, all the design-based uncertainty is captured in the expression above and we can calculate the variance of the estimator using standard expressions or techniques for the IPW estimators, but now the target estimator is for the total of the variable *u*_*rmf*_. Let *s*_R_, $s_{{\text{M}}_{r}}$ and $s_{{\text{F}}_{rm}}$ denote the sample of regions, municipalities in region *r* and facilities in municipality *m* in region *r*, respectively. Note that the variance of the estimator can be calculated using the expression

$$\begin{array}{c} Var(\hat{T})=E[Var(\hat{T}|u)]+Var[E(\hat{T}|u)]. \end{array}$$ (14)

Following [18], the expression for the first term is

$$\begin{array}{c} E[Var(\hat{T}|u)]=E[\sum_{r=1}^{N_{\text{R}}}\sum_{s=1}^{N_{\text{R}}}\Delta_{{\text{R}}_{rs}}{\overset{ˇ}{t}}_{R_{s}}{\overset{ˇ}{t}}_{R_{r}}+\sum_{r=1}^{N_{\text{R}}}w_{R_{r}}V_{R_{r}}], \end{array}$$ (15)

where $\Delta_{Rrs}=\pi_{{\text{R}}_{rs}}-\pi_{R_{r}}\pi_{R_{s}}$, $\pi_{{\text{R}}_{rs}}=P(I_{{\text{R}}_{r}}=1,I_{R_{s}}=1)$ which represents the pairwise inclusion probabilities at the regional level, ${\overset{ˇ}{t}}_{R_{r}}=w_{R_{r}}\sum_{m=1}^{N_{{\text{F}}_{r}}}\sum_{f=1}^{N_{{\text{F}}_{rm}}}u_{rmf}$. The term $V_{R_{r}}$ is given by $V_{R_{r}}=Var({\hat{t}}_{R_{r}}|u)$, where ${\hat{t}}_{R_{r}}=\sum_{m=1}^{N_{{\text{M}}_{r}}}\sum_{f=1}^{N_{F_{mr}}}I_{{\text{F}}_{rmf}}w_{{\text{F}}_{rmf}}\pi_{R_{r}}u_{rmf}$. The term $V_{R_{r}}$ is calculated in the same way as (15) and so on for each sampling stage. Further details are given in the supporting information S1. An estimator of (15) is given by

$$\begin{array}{c} \hat{E}[Var(\hat{T}|u)]=\sum_{r=1}^{N_{\text{R}}}\sum_{s=1}^{N_{\text{R}}}I_{R_{s}}I_{{\text{R}}_{r}}\frac{\Delta_{{\text{R}}_{rs}}}{\pi_{{\text{R}}_{rs}}}\frac{{\hat{t}}_{R_{s}}}{\pi_{R_{s}}}\frac{{\hat{t}}_{R_{r}}}{\pi_{R_{r}}}+\sum_{r=1}^{N_{\text{R}}}I_{R_{r}}w_{R_{r}}{\hat{V}}_{R_{r}}, \end{array}$$ (16)

where ${\hat{V}}_{R_{r}}$ is such that $E[{\hat{V}}_{R_{r}}|u,s_{\text{R}}]=V_{R_{r}}$. This expression is calculated in a similar way to (16). Details on this derivations can be found in [18]. We also provide specific formulas for (15) and (16) for the Brazilian design. And, an unbiased estimator for the second term in 14 is given by

$$\begin{array}{c} \hat{Var}[E(\hat{T}|u)]=\sum_{r=1}^{N_{\text{R}}}I_{R_{r}}\sum_{m=1}^{N_{{\text{M}}_{r}}}I_{{\text{M}}_{rm}}\frac{N_{\text{R}}N_{{\text{M}}_{r}}}{n_{\text{R}}n_{{\text{M}}_{r}}}N_{F_{mr}}{\hat{S}}_{u_{|mr}}^{2}, \end{array}$$ (17)

where

$$\begin{array}{c} {\hat{S}}_{u_{|mr}}^{2}=\frac{1}{n_{F_{mr}}-1}\sum_{f=1}^{N_{F_{mr}}}I_{{\text{F}}_{rmf}}{\left(u_{rmf}-{\tilde{u}}_{|m_{r}}\right)}^{2};\;{\tilde{u}}_{|m_{r}}=\frac{1}{n_{F_{mr}}}\sum_{f=1}^{N_{F_{mr}}}I_{{\text{F}}_{rmf}}u_{rmf}. \end{array}$$ (18)

### Estimation of the cost per dose

The cost per dose is defined as the total cost of implementation divided by the number of doses provided by the program. Mathematically, $c_{p}=\frac{T}{T_{d}}$, where *T* is the total cost of the program and $T_{d}=\sum_{r=1}^{N_{\text{R}}}\sum_{m=1}^{N_{{\text{M}}_{r}}}\sum_{f=1}^{N_{F_{mr}}}d_{rmf}$ is the total number of doses, with *d*_*rmf*_ being the number of doses in facility *f* in municipality *m* in region *r*. The estimate of the cost per dose is

$$\begin{array}{c} {\hat{c}}_{p}=\frac{\hat{T}}{T_{d}}, \end{array}$$ (19)

where $\hat{T}$ is the estimated total cost obtained in (13). The variance of this estimator is given by

$$\begin{array}{c} Var\left({\hat{c}}_{p}\right)=\frac{Var(\hat{T})}{T_{d}^{2}} \end{array}$$ (20)

and an unbiased estimator of this quantity is given by

$$\begin{array}{c} \hat{Var}({\hat{c}}_{p})=\frac{\hat{V}(\hat{T})}{T_{d}^{2}}, \end{array}$$ (21)

where $\hat{V}\left(\hat{T}\right)$ is given by (14).

## Post-design methods: Calibrated weights

The main purpose of post-designs methods is to improve the estimates by reducing the variance, i. e. to produce more efficient estimators. Post-design methods include techniques such as post stratification, estimation of weights and calibration of weights. The latter is known to yield more efficient estimates by adjusting the weights using information readily-available at first phase. In Brazil, for example, the total number of doses administered and the total number of facilities in the country is known. Hence this can be used to adjust (calibrate) the weights.

[13] proposed the use of calibration to adjust the sampling weights in case–cohort designs. Let *v*_F_, *v*_M_ and *v*_R_ denote readily available variables at first phase and at the different levels. The former is a variable available for all units at the final stage, the second is available for all second stage units and the latter is available for all first-stage units, e.g regions. The main goal is to adjust weights such that estimates for the totals ${\hat{T}}_{v_{\text{F}}}$, ${\hat{T}}_{v_{\text{M}}}$ and ${\hat{T}}_{v_{\text{R}}}$ equal the actual totals $T_{v_{\text{F}}}$, $T_{v_{\text{M}}}$ and $T_{v_{\text{R}}}$, but forcing the new weights ($\hat{w}$) to be as close as possible to the design weights. For example, consider IPW estimates ${\hat{T}}_{v_{\text{F}}}$ and ${\hat{T}}_{v_{\text{M}}}$ based on the same weights used to estimate *T* with $\hat{T}$, i.e.

$$\begin{array}{c} {\hat{T}}_{v_{\text{F}}}=\sum_{s_{{\text{F}}_{rm}}}w_{{\text{F}}_{rmf}}{v_{\text{F}}}_{rmf};\;{\hat{T}}_{v_{\text{M}}}=\sum_{s_{{\text{M}}_{r}}}w_{{\text{M}}_{rm}}{v_{\text{M}}}_{rm};\;{\hat{T}}_{v_{\text{R}}}=\sum_{s_{\text{R}}}w_{{\text{R}}_{r}}{v_{\text{R}}}_{r}. \end{array}$$

Since we know $T_{v_{\text{F}}}$, $T_{v_{\text{M}}}$ and $T_{v_{\text{R}}}$ we can slightly modify the weights (*w*) and find ($\hat{w}$) such that

$$\begin{array}{ccc} {\hat{T}}_{v_{\text{F}}} & = & \sum_{s_{{\text{F}}_{rm}}}{\hat{w}}_{{\text{F}}_{rmf}}{v_{\text{F}}}_{rmf}=T_{v_{\text{F}}};\\ {\hat{T}}_{v_{\text{M}}} & = & \sum_{s_{{\text{M}}_{r}}}{\hat{w}}_{{\text{M}}_{rm}}{v_{\text{M}}}_{rm}=T_{v_{\text{M}}};\\ {\hat{T}}_{v_{\text{R}}} & = & \sum_{s_{\text{R}}}{\hat{w}}_{{\text{R}}_{r}}{v_{\text{R}}}_{r}=T_{v_{\text{R}}}. \end{array}$$ (22)

Intuitively, the modified weights (${\hat{w}}_{{\text{F}}_{rmf}},{\hat{w}}_{{\text{M}}_{rm}}$) contain information about the entire population of facilities and we can use the new weights to find a new estimate of *T*:

$$\begin{array}{c} {\hat{T}}_{cal}={\hat{T}}_{\text{F},cal}+{\hat{T}}_{\text{M},cal}+{\hat{T}}_{\text{R},cal}+T_{\text{C}}=\sum_{s_{{\text{F}}_{rm}}}{\hat{w}}_{{\text{F}}_{rmf}}y_{{\text{F}}_{rmf}}+\sum_{s_{{\text{M}}_{r}}}{\hat{w}}_{{\text{M}}_{rm}}y_{{\text{M}}_{rm}}+\sum_{s_{\text{R}}}{\hat{w}}_{{\text{R}}_{r}}y_{{\text{R}}_{r}}+T_{\text{C}}, \end{array}$$

where ${\hat{T}}_{\text{F},cal},{\hat{T}}_{\text{M},cal}$ and ${\hat{T}}_{\text{R},cal}$ represent the estimators of total cost at the Facility, Municipality and regional level, respectively. Mathematically, a distance function $d(w,\hat{w})$ measuring the distance from original weights(*w*) to new weights($\hat{w}$) is minimized subject to the constrains (22). There are several options for choosing this distance. Substantial gains in accuracy depend on how correlated the calibration variables are with the quantity of interest [9].

### Variance estimation

In order to estimate the variance in practice we use a Taylor expansion as in [18]. Define

$$\begin{array}{c} {\tilde{u}}_{rmf}=e_{{\text{F}}_{rmf}}+\frac{w_{{\text{M}}_{rm}}}{w_{{\text{F}}_{rmf}}n_{F_{mr}}}e_{{\text{M}}_{rm}}+\frac{w_{{\text{R}}_{r}}}{w_{{\text{F}}_{rmf}}n_{{\text{F}}_{r}}}e_{{\text{R}}_{r}}, \end{array}$$

where $e_{{\text{F}}_{rmf}}$, $e_{{\text{M}}_{rm}}$ and $e_{{\text{R}}_{r}}$ y are given by

$$\begin{array}{c} \begin{array}{cc} & {e_{\text{F}}}_{rmf}={y_{\text{F}}}_{rmf}-{v_{\text{F}}}_{rmf}^{\text{T}}{\tilde{B}}_{\text{F}};\;{\tilde{B}}_{\text{F}}={\tilde{T}}_{\text{F}}^{-1}(\sum_{r=1}^{N_{\text{R}}}\sum_{m=1}^{N_{{\text{M}}_{r}}}\sum_{f=1}^{N_{F_{m_{r}}}}v_{{\text{R}}_{rmf}})\\ & {e_{\text{M}}}_{rm}={y_{\text{M}}}_{rm}-{v_{\text{M}}}_{rm}^{\text{T}}{\tilde{B}}_{\text{M}};\;{\tilde{B}}_{\text{M}}={\tilde{T}}_{\text{M}}^{-1}(\sum_{r=1}^{N_{\text{R}}}\sum_{m=1}^{N_{{\text{M}}_{r}}}v_{{\text{M}}_{rm}})\\ & {e_{\text{R}}}_{r}={y_{\text{R}}}_{r}-{v_{\text{R}}}_{r}^{\text{T}}{\tilde{B}}_{\text{R}};\;{\tilde{B}}_{\text{R}}={\tilde{T}}_{\text{R}}^{-1}(\sum_{r=1}^{N_{\text{R}}}\sum_{m=1}^{N_{{\text{M}}_{r}}}v_{{\text{R}}_{rm}}), \end{array} \end{array}$$ (23)

and

$$\begin{array}{c} \begin{array}{cc} & {\tilde{w}}_{{\text{F}}_{rmf}}=w_{{\text{F}}_{rmf}}(1+v_{{\text{F}}_{rmf}}^{\text{T}}{\tilde{\lambda }}_{\text{R}});\;{\tilde{\lambda }}_{\text{R}}={\tilde{T}}_{\text{R}}^{-1}(T_{v_{\text{F}}}-{\hat{T}}_{v_{\text{F}}});\;{\tilde{T}}_{\text{F}}=\sum_{r=1}^{n_{\text{R}}}\sum_{m=1}^{n_{{\text{M}}_{r}}}\sum_{f=1}^{n_{F_{m_{r}}}}{\tilde{w}}_{{\text{F}}_{rmf}}v_{{\text{F}}_{rmf}}{v_{{\text{F}}_{rmf}}}^{\text{T}};\\ & {\tilde{w}}_{{\text{M}}_{rm}}=w_{{\text{F}}_{rm}}(1+v_{{\text{M}}_{rm}}^{\text{T}}{\tilde{\lambda }}_{\text{M}});\;{\tilde{\lambda }}_{\text{M}}={\tilde{T}}_{\text{M}}^{-1}(T_{v_{\text{M}}}-{\hat{T}}_{v_{\text{M}}});\;{\tilde{T}}_{\text{M}}=\sum_{r=1}^{n_{\text{R}}}\sum_{m=1}^{n_{{\text{M}}_{r}}}{\tilde{w}}_{{\text{M}}_{rm}}v_{{\text{M}}_{rm}}{v_{{\text{M}}_{rm}}}^{\text{T}};\\ & {\tilde{w}}_{{\text{R}}_{r}}=w_{{\text{R}}_{r}}(1+v_{{\text{R}}_{r}}^{\text{T}}{\tilde{\lambda }}_{\text{R}});\;{\tilde{\lambda }}_{\text{F}}={\tilde{T}}_{\text{F}}^{-1}(T_{v_{\text{F}}}-{\hat{T}}_{v_{\text{F}}});\;{\tilde{T}}_{\text{R}}=\sum_{r=1}^{n_{\text{R}}}{\tilde{w}}_{{\text{R}}_{r}}v_{{\text{R}}_{r}}{v_{{\text{R}}_{r}}}^{\text{T}}. \end{array} \end{array}$$ (24)

Therefore

$$\begin{array}{c} \hat{Var}({\hat{T}}_{cal})=Var({\hat{T}}_{e})=\hat{E}[Var({\hat{T}}_{e}|u)]+\hat{Var}[E({\hat{T}}_{e}|u)], \end{array}$$ (25)

with ${\hat{T}}_{e}=\sum_{r=1}^{N_{\text{R}}}\sum_{m=1}^{N_{{\text{M}}_{r}}}\sum_{f=1}^{N_{F_{mr}}}I_{{\text{R}}_{r}}I_{{\text{M}}_{rm}}I_{{\text{F}}_{rmf}}w_{{\text{F}}_{rmf}}{\tilde{u}}_{rmf}$. Expression (25) is found in a similar way as the variance estimate in section Variance estimation.

## Results from Brazilian study

Table 2 displays the information used for calibration of the weights. At the facility level, weights (*w*_*F*_) were calibrated using all the totals the totals presented in Table 2. This includes information on the facility size, the total number of doses in 2014 and the number of facilities in each region. Furthermore, at the municipality level, weights(*w*_*M*_) were calibrated using only the total number of municipalities. The number of expected municipalities in the sample was 55, but the actual sample contained 38 municipalities. This was due to sampling variation and due to two missing municipalities.

**Table 2:** Information used for calibration of the weights. FAcility size is defined as follows. Huge (No.Doses > 10000); large (5000<No.Doses ≤ 10000); medium (1500<No.Doses ≤ 5000); small (500 < No.Doses ≤ 1500) and tiny (No.Doses ≤ 500).

	Huge	Large	Medium	Small	Tiny	Total Doses
Frame	1727	3210	10917	8260	3273	89928432
Sample	77	61	106	47	34	2822574
	Midwest	Northeast	North	Southeast	South	No. Municipalities
Frame	1988	10897	2103	8312	4087	5543
Sample	64	64	65	66	66	38

Tables 3 and 4 display the estimated total cost for each of the different categories of cost at the municipal level and for the entire program, respectively. Table 3 presents the estimates of costs incurred by municipalities. The data used to estimate this was the sample of 38 municipalities. The table shows significant differences between the estimates using the unadjusted sampling weights and the estimates using the calibrated weights. The reason for this is the small sample of municipalities available. With this in mind, adjusting/ calibrating the sampling weights yields more representative and informative estimators. In addition, due to the increase in magnitude, the SE are larger for the calibrated estimators.

Table 4 displays the estimates of the total cost of the entire program for each of the different categories. As observed, the estimates differ in magnitude for all the categories and the calibrated estimates are always larger. An additional feature of the calibrated estimates is that their standard errors are lower than those obtained with the unadjusted sampling weights. This leads to the conclusion that the calibrated estimates are more precise and efficient and therefore more reliable.

### Results

**Table 3:** Estimated municipality level costs, by cost category, considering unadjusted and calibrated weight (in million R$) for the Costing Study of the Brazilian Immunization Program. Brazil, 2013. Standard errors are displayed in parenthesis. Municipal level weights were calibrated to the national number of municipalities.

Category	Sampling weights			Calibrated weights		
	Capital	Recurrent	Total	Capital	Recurrent	Total
Vehicles	43(14)	4.3(1.4)	47(15.1)	202 (71)	20.2(7.1)	222(78.1)
Equipment	3.8(0.6)	0.4(0.1)	4.2(0.7)	18.1(4.7)	1.8(0.5)	20(5.2)
Buildings	9.9(1.7)	72(44.7)	82.1(45)	47(12.9)	341(233)	387(239)
Labor		125(21.6)	126(22)		593(123)	593(123)
Other		12(5.7)	12(5.7)		56.5(29.5)	56.5(29.5)
Total	57(14.8)	215(51.5)	271(54)	267(82)	1012(314)	1279(355)

**Table 4:** Estimated total costs, by cost category, considering unadjusted and calibrated weight (in million R$) for the Costing Study of the Brazilian Immunization Program. Brazil, 2013. Standard errors are displayed in parenthesis. This includes facility level cost, municipality level cost and state level cost. Municipal level weights were calibrated to the national number of municipalities, and facility level weights were calibrated using information presented in Table 2.

Category	Sampling weights			Calibrated weights		
	Capital	Recurrent	Total	Capital	Recurrent	Total
Vehicles	45.1 (6.8)	8.5 (1.1)	53.5 (7.8)	248 (50.3)	27.8 (5.2)	275.8 (55.5)
Equipment	25.6 (3.4)	4.2 (0.6)	29.8 (4)	46.3 (5.9)	6.3 (0.8)	52.6 (6.6)
Buildings	83.1 (8.9)	94.7 (32.3)	177.8 (35.2)	137.5 (12.2)	466 (165.8)	604 (169.3)
Labor		1310 (122.2)	1310 (122.2)		1956 (186.2)	1956(186.2)
Other		16.9 (3.4)	16.9 (3.4)		107.1 (34.3)	107.1 (34.3)
Total	153.8 (16.2)	1434 (136.3)	1588 (149.8)	431.8 (60.8)	2564 (291.4)	2996 (331.4)

## Discussion

We have proposed inverse probability weighting(IPW) with calibration methods to improve estimators in studies where information is collected at several levels, e.g. states-municipalities-clinics. We find an expression for the estimator of the total such that it accounts for the totals at each level and also provides a feasible way to find the variance. Further, we use information available for all units in the targeted population to adjust the weights with calibration methods. It allows for calibration of the weights at all the levels of sampling if desired by investigators. These methods can be used to correct for missing information at the various levels. This is achieved by adjusting the estimates at each level to comply with the characteristics of the country. The methods were applied to the Brazilian National Immunization Program. The results showed that calibrating the weights does not only correct for missingness, but also results in large gains in efficiency and precision compared to standard methods. The estimators result difficult to compare in terms of efficiency due to the difference in magnitude. To provide an interpretation, we considered that the estimates obtained by calibration were consistent, which is supported by asymptotic theory [9, 10]. We compare the estimated MSEs as follows. For the total cost with standard weights, the MSEs are: 278(Capital), 1138(Recurrent), 1415(Total). With calibrated weights these are 61(Capital), 291 (Recurrent), 331(Total). Calibration clearly returns smallest MSEs if the estimator is consistent (or unbiased). A point of interest for future research is to investigate how different would the results have been if the sampling design had been different. For example, stratifying by facility size and/or type. This can enhance estimators because facility size/type is related to the cost. On the contrast, stratification by something not related to the cost would not yield gains in efficiency. We hope that investigators become more aware of the existence and implementation of these methods. This can help analyst and policy-makers to inform on the basis of more reliable information.

## Supporting information

S1 Appendix Estimation and variance of the total cost and cost per dose from the Brazilian immunization study. (PDF)

S2 Appendix Estimation and variance of the total cost and cost per dose using design and calibrated weights. (PDF)

S1 Dataset The dataset contains the data for the estimation of the total cost from the Brazilian study. (TXT)
