# Correction: Improved calibration estimators for the total cost of health programs and application to immunization in Brazil

**DOI:** 10.1371/journal.pone.0215701

**Published:** 2019-04-15

**Authors:** Claudia Rivera-Rodriguez, Cristiana Toscano, Stephen Resch

The following information is missing from the Funding statement: This study was funded by the Brazilian Ministry of Health, with additional support provided by the Pan-American Health Organization and the Bill & Melinda Gates Foundation.
