# Supplementary material for: Improved calibration estimators for the total cost of health programs and application to immunization in Brazil
Source: PLoS One. 2019 Mar 6;14(3):e0212401. doi: 10.1371/journal.pone.0212401 (PMC6402677; doi:10.1371/journal.pone.0212401)
Supplement: S1 Appendix — (PDF) [file pone.0212401.s001.pdf]

S1 Appendix. Estimation and variance of the total cost and cost per dose from the Brazilian immunization study.

c.rodriguez@auckland.ac.nz, stclr@channing.harvard.edu

February 14, 2019

1 Results from the Brazilian immunization study

Tables 1 2 and 3 display the estimated total cost and standard errors for the immunization project for each of the different categories and types of cost. Table 2 presents the estimated totals while table 3 shows the estimated standard errors. The tables contain estimates with both unadjusted and calibrated weights. At the facility level, weights(w_F) were calibrated using the totals presented in table 1. This includes information on the facility size, the total number of doses in 2014 and the number of facilities in each region. At the municipality level, weights(w_M) were only calibrated using the total number of municipalities. The number of expected municipalities in the sample was 55, but there were only 37. This was in part due to sampling variation and also due to two missing municipalities. As observed in table 2, the estimates differ significantly using unadjusted and calibrated weights. This was caused by the missing municipalities, which implied that there were also missing facilities.

As observed in table 3, an additional feature of the calibrated estimates is that their standard errors are much lower that the ones obtained with unadjusted sampling weights. This means that the calibrated estimates are more accurate and therefore more reliable.These results could be used with more confidence when informing policy and planning.

Table 1: Information used for calibration of the weights at the facility level (W_F): Total number of facilities in each category, the first row represents the totals $\mathbf{T}_{\mathrm{F}}$.

	Huge	Large	Medium	Small	Tiny	Total Doses	Midwest	Northeast	North	Southeast	South
Frame ($\mathbf{T}_{\mathbf{F}}$)	1728	3216	10928	8267	3248	90051490	1988	10897	2103	8312	4087
Sample	77	62	112	47	27	2847025	64	64	65	66	66

Table 2: Total cost estimates using unadjusted and calibrated weights weights

Category	Area	Total Capital Cost		Total Recurrent Cost		Total Cost	
		Unadjust.	Calib.	Unadjust.	Calib.	Unadjust.	Calib.
VehAir	CC	0.00	0.00	0.00	0.00	0.00	0.00
VehAir	PNI	0.00	0.00	0.00	0.00	0.00	0.00
VehAir	Regionais	0.00	0.00	0.00	0.00	0.00	0.00
Vehicles	CC	1.25	1.25	2.41	2.41	3.65	3.65
Vehicles	PNI	43.97	204.09	6.07	22.08	50.03	226.17
Vehicles	Regionais	0.00	0.00	0.00	0.00	0.00	0.00
Equipment	CC	3.57	13.20	0.36	1.32	3.93	14.52
Equipment	PNI	18.27	26.92	3.51	4.78	21.79	31.70
Equipment	Regionais	3.73	3.73	0.37	0.37	4.11	4.11
Buildings	CC	7.14	15.61	9.97	14.65	17.12	30.27
Buildings	PNI	75.95	122.69	37.40	127.09	113.35	249.77
Buildings	Regionais	0.00	0.00	0.00	0.00	0.00	0.00
Labor	CC	0.00	0.00	31.85	122.20	31.85	122.20
Labor	PNI	0.00	0.00	1209.55	1907.79	1209.55	1907.79
Labor	Regionais	0.00	0.00	55.51	55.51	55.51	55.51
Vaccines	CC	0.00	0.00	0.00	0.00	0.00	0.00
Vaccines	PNI	0.00	0.00	0.00	0.00	0.00	0.00
Vaccines	Regionais	0.00	0.00	0.00	0.00	0.00	0.00
Other	CC	0.00	0.00	0.00	0.00	0.00	0.00
Other	PNI	0.00	0.00	16.88	61.49	16.88	61.49
Other	Regionais	0.00	0.00	0.00	0.00	0.00	0.00
		0.00	0.00	0.00	0.00	0.00	0.00
VehAir		0.00	0.00	0.00	0.00	0.00	0.00
Vehicles		45.21	205.34	8.48	24.49	53.69	229.83
Equipment		25.58	43.85	4.24	6.47	29.82	50.32
Buildings		83.10	138.30	47.37	141.74	130.47	280.04
Labor		0.00	0.00	1296.91	2085.50	1296.91	2085.50
Vaccines		0.00	0.00	0.00	0.00	0.00	0.00
Other		0.00	0.00	16.88	61.49	16.88	61.49
		0.00	0.00	0.00	0.00	0.00	0.00
	CC	11.96	30.06	44.59	140.58	56.55	170.64
	PNI	138.19	353.70	1273.40	2123.22	1411.59	2476.92
	Regionais	3.73	3.73	55.88	55.88	59.61	59.61
Total	Total	153.88	387.49	1373.87	2319.69	1527.75	2707.18

2

Table 3: Standard errors estimates using unadjusted and calibrated weights weights

Category	Area	SE Total Capital Cost		SE Total Recurrent Cost		SE Total Cost	
		Unadjust.	Calib.	Unadjust.	Calib.	Unadjust.	Calib.
VehAir	CC	0.0	0.0	0.0	0.0	0.0	0.0
VehAir	PNI	0.0	0.0	0.0	0.0	0.0	0.0
VehAir	Regionais	0.0	0.0	0.0	0.0	0.0	0.0
Vehicles	CC	0.2	0.0	0.3	0.0	0.5	0.0
Vehicles	PNI	6.6	24.6	0.8	2.5	7.5	27.0
Vehicles	Regionais	0.0	0.0	0.0	0.0	0.0	0.0
Equipment	CC	0.5	1.7	0.0	0.2	0.5	1.9
Equipment	PNI	2.6	2.2	0.5	0.4	3.1	2.6
Equipment	Regionais	0.5	0.0	0.0	0.0	0.6	0.0
Buildings	CC	0.9	1.5	1.3	1.5	2.1	2.6
Buildings	PNI	8.2	7.9	4.2	13.1	11.6	17.1
Buildings	Regionais	0.0	0.0	0.0	0.0	0.0	0.0
Labor	CC	0.0	0.0	4.9	16.2	4.9	16.2
Labor	PNI	0.0	0.0	113.2	116.6	113.2	116.6
Labor	Regionais	0.0	0.0	7.5	0.0	7.5	0.0
Vaccines	CC	0.0	0.0	0.0	0.0	0.0	0.0
Vaccines	PNI	0.0	0.0	0.0	0.0	0.0	0.0
Vaccines	Regionais	0.0	0.0	0.0	0.0	0.0	0.0
Other	CC	0.0	0.0	0.0	0.0	0.0	0.0
Other	PNI	0.0	0.0	3.4	12.2	3.4	12.2
Other	Regionais	0.0	0.0	0.0	0.0	0.0	0.0
		0.0	0.0	0.0	0.0	0.0	0.0
VehAir		0.0	0.0	0.0	0.0	0.0	0.0
Vehicles		6.8	24.6	1.1	2.5	7.8	27.0
Equipment		3.4	3.4	0.6	0.5	4.0	3.9
Buildings		8.9	8.2	5.2	13.1	13.4	17.1
Labor		0.0	0.0	121.9	116.6	121.9	116.6
Vaccines		0.0	0.0	0.0	0.0	0.0	0.0
Other		0.0	0.0	3.4	12.2	3.4	12.2
		0.0	0.0	0.0	0.0	0.0	0.0
	CC	1.4	2.8	6.0	15.6	7.3	17.4
	PNI	14.4	25.6	117.6	120.6	129.8	131.9
	Regionais	0.5	0.0	7.5	0.0	8.0	0.0
Total	Total	16.2	26.4	127.8	120.5	141.9	132.2

REFERENCES
