# Supplementary material for: Improved calibration estimators for the total cost of health programs and application to immunization in Brazil
Source: PLoS One. 2019 Mar 6;14(3):e0212401. doi: 10.1371/journal.pone.0212401 (PMC6402677; doi:10.1371/journal.pone.0212401)
Supplement: S2 Appendix — (PDF) [file pone.0212401.s002.pdf]

S2 Appendix. Estimation and variance of the total cost and cost per dose using design and calibrated weights.

c.rodriguez@auckland.ac.nz, stclr@channing.harvard.edu

February 14, 2019

1 Estimation of total cost T

The scientific goal is the estimation of a total. This total is composed of totals at different levels. For example, for the Brazilian immunization study there are costs that are specific to the regions, some other costs are specific to the municipalities and some others are specific to the facilities. We introduce the following notation in order to develop the details for a three multi-stage design.

Let $y_{\mathrm{F}rmf}$ be the value specific to health facility f, in municipality m, in r.

Let $y_{\mathrm{M}rm}$ represents the value specific to municipality m, in region r.

Let $y_{\mathrm{R}r}$ represents the value specific to region r.

In addition, let U_{R} be the set of all regions, U_{M_r} be the set of all municipalities in region r and $U_{F_{mr}}$ be the set of all facilities in municipality m, region r. There are in total N_{R} regions, N_{M_r} municipalities in region r and $N_{F_{mr}}$ facilities in municipality m in region r. The total cost can be represented as

$$T = T_{\mathrm{C}} + T_{\mathrm{F}} + T_{\mathrm{M}} + T_{\mathrm{R}} = T_{\mathrm{C}} + \sum_{r=1}^{N_{\mathrm{R}}} \sum_{m=1}^{N_{\mathrm{M}_r}} \sum_{f=1}^{N_{F_{mr}}} y_{\mathrm{F}rmf} + \sum_{r=1}^{N_{\mathrm{R}}} \sum_{m=1}^{N_{\mathrm{M}_r}} y_{\mathrm{M}rm} + \sum_{r=1}^{N_{\mathrm{R}}} y_{\mathrm{R}r}. \tag{1}$$

where T_{C} represents the additional cost accrued centrally and it is assumed known.

1.1 The design

To estimate (1), a multi-stage simple sampling design is used. Let s_{R} represent the sub sample of regions, s_{M_r} represent the sub sample of municipalities in region r and $s_{F_{mr}}$ represent the sub sample facilities in municipality m in region r, respectively. The design is as follows. At first stage, a simple sample, s_{R}, of n_{R} regions is selected from U_{R} with replacement. At second stage, for each region in s_{R}, a sample of n_{M_r} municipalities is selected from each U_{M_r}. Finally, at third stage, within each municipality in s_{M_r}, a sample of $n_{F_{mr}}$ facilities is selected from $U_{F_{mr}}$.

Each facility has a probability of being included in the final sample. This design can be characterize by its sampling probabilities. These probabilities are used in the estimation of the total T. This sampling probability for facility f from municipality m, from region r, can be written as

$$\begin{aligned} \pi_{\mathrm{F}rmf} &= P(r \in s_{\mathrm{R}}, m \in s_{\mathrm{M}_r}, f \in s_{F_{mr}}) \\ \pi_{\mathrm{F}rmf} &= P(f \in s_{F_{mr}} | r \in s_{\mathrm{R}}, m \in s_{\mathrm{M}_r}) P(m \in s_{\mathrm{M}_r} | r \in s_{\mathrm{R}}) P(r \in s_{\mathrm{R}}) \\ &= \pi_{f|rm} \pi_{m|r} \pi_{\mathrm{R}r} = \frac{n_{F_{mr}}}{N_{F_{mr}}} \frac{n_{\mathrm{M}_r}}{N_{\mathrm{M}_r}} \frac{n_{\mathrm{R}}}{N_{\mathrm{R}}}. \end{aligned} \tag{2}$$

These probabilities can be written in this form only if we can assume that the sampling designs within each region was planned before the regions were selected and that the sampling design was implemented as planned, then we have a principle called *invariance*. Otherwise, it is not guaranteed that the probabilities can be calculated. Note that

$$I_{\mathrm{F}_{rmf}} = I_{\mathrm{F}_{rmf}} I_{\mathrm{M}_{rm}} I_{\mathrm{R}_r}; \quad I_{\mathrm{M}_{rm}} = I_{\mathrm{M}_{rm}} I_{\mathrm{R}_r}, \tag{3}$$

where $I_{\mathrm{F}_{rmf}}, I_{\mathrm{M}_{rm}}$ and I_{R_r} respectively represent the inclusion indicators for region r, municipality m in region r and faiclity f in municipality m in region r. The conditional inclusion probabilities are defined as $\pi_{f|mr} = E\left(I_{\mathrm{F}_{rmf}=1} \middle| I_{\mathrm{M}_{rm}=1}\right) = \frac{n_{F_{mr}}}{N_{F_{mr}}}$; $\pi_{\mathrm{R}r} = E\left(I_{\mathrm{R}_r=1}\right) = \frac{n_{\mathrm{R}}}{N_{\mathrm{R}}}$ and $\pi_{m|r} = E\left(I_{\mathrm{M}_{rm}=1} \middle| I_{\mathrm{R}_r=1}\right) = \frac{n_{\mathrm{M}_r}}{N_{\mathrm{M}_r}}$. Using this, the number of facilities sampled in municipality, region r can be written as $n_{\mathrm{F}_{rm}} = \sum_{f=1}^{N_{\mathrm{F}_{rm}}} I_{\mathrm{F}_{rmf}}$ and the number of facilities sampled in region r can be written as $n_{\mathrm{F}_r} = \sum_{m=1}^{N_{\mathrm{F}_r}} I_{\mathrm{M}_{rm}} \sum_{f=1}^{N_{\mathrm{F}_{rm}}} I_{\mathrm{F}_{rmf}} I_{\mathrm{F}_{rmf}}$ and these quantities are fixed by design. Likewise, let $N_{\mathrm{F}_{rm}}$ be the number of facilities in municipality, region r and N_{F_r} be the number of facilities sampled in region r.

1.2 Estimating T_{F}

We now present the estimation of the total and its standard error in the next sections.

- Under the design above, the weighted estimator of T_{F} is given by

$$\widehat{T}_{\text{F}} = \sum_{r=1}^{n_{\text{R}}} \sum_{m=1}^{n_{\text{M}_r}} \sum_{f=1}^{n_{F_{mr}}} w_{\text{R}rmf} y_{\text{R}rmf} = \sum_{r=1}^{N_{\text{R}}} \sum_{m=1}^{N_{\text{M}_r}} \sum_{f=1}^{N_{F_{mr}}} I_{\text{F}_{rmf}} w_{\text{F}_{rmf}} y_{\text{F}_{rmf}}, \tag{4}$$

where each $w_{\text{R}rmf}$ represents the sampling weight for facility f from municipality m, from region r.

- These weights are given by

$$w_{\text{R}rmf} = 1/\pi_{\text{R}rmf} = \frac{1}{\pi_{f|mr}\pi_{m|r}\pi_{\text{R}r}},$$

where $\pi_{\text{R}rmf}$ is the inclusion probability for facility f in municipality m in region r.

1.3 Estimating T_{M}

- Let $w_{\text{M}rm} = \frac{1}{\pi_{m|r}\pi_{\text{R}_r}}$; that is, the weight for municipality m, which belongs to region r.
- The weighted estimator of T_{M} is given by

$$\begin{aligned}
\widehat{T}_{\text{M}} &= \sum_{r=1}^{n_{\text{R}}} \sum_{m=1}^{n_{\text{M}_r}} w_{\text{M}rm} y_{\text{M}rm} = \sum_{r=1}^{n_{\text{R}}} \sum_{m=1}^{n_{\text{M}_r}} \sum_{f=1}^{n_{F_{mr}}} w_{\text{F}rmf} \frac{w_{\text{M}rm}}{w_{\text{F}rmf} n_{F_{mr}}} y_{\text{M}_{rm}} \\
&= \sum_{r=1}^{N_{\text{R}}} \sum_{m=1}^{N_{\text{M}_r}} I_{\text{M}_{rm}} w_{\text{M}_{rm}} y_{\text{M}_{rm}} = \sum_{r=1}^{N_{\text{R}}} I_{\text{R}_r} \sum_{m=1}^{N_{\text{M}_r}} I_{\text{M}_{rm}} \sum_{f=1}^{N_{F_{mr}}} I_{\text{F}_{rmf}} \frac{w_{\text{F}_{rmf}} w_{\text{M}_{rm}} y_{\text{M}_{rm}}}{w_{\text{F}_{rmf}} n_{F_{mr}}}.
\end{aligned} \tag{5}$$

This estimator is unbiased for T_{M}. The reason why we express (5) in terms of three sums is because it is used to calculate the variance.

1.4 Estimating T_{R}

- Let $w_{\text{R}_r} = \frac{1}{\pi_{\text{R}_r}}$, that is, the weight corresponding to region r.
- The weighted estimator of T_{R} is given by

$$\begin{aligned}
\widehat{T}_{\text{R}} &= \sum_{r=1}^{n_{\text{R}}} w_{\text{R}_r} y_{\text{R}_r} = \sum_{r=1}^{n_{\text{R}}} \sum_{m=1}^{n_{\text{M}_r}} \sum_{f=1}^{n_{F_{mr}}} w_{\text{F}_{rmf}} \frac{w_{R_r}}{w_{\text{F}_{rmf}} n_{\text{F}_r}} y_{\text{R}_r} \\
&= \sum_{r=1}^{N_{\text{R}}} I_{\text{R}_r} w_{\text{R}_r} y_{\text{R}_r} = \sum_{r=1}^{N_{\text{R}}} I_{\text{R}_r} \sum_{m=1}^{N_{\text{M}_r}} I_{\text{M}_{rm}} \sum_{f=1}^{N_{F_{mr}}} I_{\text{F}_{rmf}} \frac{w_{\text{F}_{rmf}} w_{R_r} y_{\text{R}_r}}{w_{\text{F}_{rmf}} n_{\text{F}_r}}.
\end{aligned} \tag{6}$$

This estimator is unbiased for T_{R}.

1.5 The estimator of the total

Usually, IPW estimators only involve data at the last stage of the design, such as $\widehat{T}_{\text{F}}$. In this case, the variance of the estimator is well known and straight forward to calculate following standard properties of multi stage designs Sarndal et al. [1992]. Our estimator of interest, however, involves information at every stage of the design. In order to find an expression for the variance of $\widehat{T}$, we define the variables:

$$u_{rmf} = y_{\text{F}_{rmf}} + \frac{w_{\text{M}_{rm}}}{w_{\text{F}_{rmf}} n_{F_{mr}}} y_{\text{M}_{rm}} + \frac{w_{\text{R}_r}}{w_{\text{F}_{rmf}} n_{\text{F}_r}} y_{\text{R}_r} + \frac{T_{\text{C}}}{n}. \tag{7}$$

$$u^*_{rmf} = y_{\text{F}_{rmf}} + \frac{1}{N_{F_{mr}}} y_{\text{M}_{rm}} + \frac{1}{N_{\text{F}_r}} y_{\text{R}_r} + \frac{T_{\text{C}}}{N}. \tag{8}$$

Then, the estimator $\widehat{T}$ be written as

$$\widehat{T} = \sum_{r=1}^{N_{\mathrm{R}}} I_{\mathrm{R}_r} \sum_{m=1}^{N_{\mathrm{M}_r}} I_{\mathrm{M}_{rm}} \sum_{f=1}^{N_{F_{mr}}} I_{\mathrm{F}_{rmf}} w_{\mathrm{F}_{rmf}} u_{rmf} \tag{9}$$

and the total can be expressed as

$$T = \sum_{r=1}^{N_{\mathrm{R}}} \sum_{m=1}^{N_{\mathrm{M}_r}} \sum_{f=1}^{N_{F_{mr}}} u^*_{rmf}. \tag{10}$$

Note that $E\left(\widehat{T}\middle| u\right) = T$.

2 Variance of $\widehat{T}$

The variance of (9) can be calculated as

$$Var\left(\widehat{T}\right) = E\left[Var\left(\widehat{T}\middle| u\right)\right] + Var\left[E\left(\widehat{T}\middle| u\right)\right], \tag{11}$$

where the first term represents the variation due to the sampling design and can be written as

$$E\left[Var\left(\widehat{T}\middle| u\right)\right] = E\left[\sum_{r=1}^{N_{\mathrm{R}}} \sum_{s=1}^{N_{\mathrm{R}}} \Delta_{\mathrm{R}_{rs}} \check{t}_{R_s} \check{t}_{\mathrm{R}_r} + \sum_{r=1}^{N_{\mathrm{R}}} w_{\mathrm{R}_r} V_{\mathrm{R}_r}\right], \tag{12}$$

where $\Delta_{Rrs} = \pi_{\mathrm{R}_{rs}} - \pi_{\mathrm{R}_r}\pi_{R_s}$, $\pi_{\mathrm{R}_{rs}} = P\left(I_{\mathrm{R}_r} = 1, I_{R_s} = 1\right)$ which represents the pairwise inclusion probabilities at the regional level, $\check{t}_{\mathrm{R}_r} = w_{\mathrm{R}_r} \sum_{m=1}^{N_{\mathrm{F}_r}} \sum_{f=1}^{N_{\mathrm{F}rm}} u_{rmf}$. The term V_{R_r} is given by $V_{\mathrm{R}_r} = Var\left(\widehat{t}_{\mathrm{R}_r}\middle| u\right)$, where $\widehat{t}_{\mathrm{R}_r} = \sum_{m=1}^{N_{\mathrm{M}_r}} \sum_{f=1}^{N_{F_{mr}}} I_{\mathrm{F}_{rmf}} w_{\mathrm{F}_{rmf}} \pi_{\mathrm{R}_r} u_{rmf}$. The term V_{R_r} is calculated in the same way as (12) . For a three multi-stage design, we can express (12) as

$$E\left[Var\left(\widehat{T}\middle| u\right)\right] = E\left[V_{\mathrm{R}}(\widehat{T}) + V_{\mathrm{M}}(\widehat{T}) + V_{\mathrm{F}}(\widehat{T})\right]. \tag{13}$$

This expression is found using conditional expectations properties, where V_{R} represents the variation due to the sampling of regions, V_{M} represents the variation due to the sampling of municipalities V_{F} is the variation due to the sampling of health facilities (see, e.g. [Sarndal et al., 1992]). Under the underlying design, they have the forms

$$V_{\mathrm{R}} = \left(\frac{1}{n_{\mathrm{R}}} - \frac{1}{N_{\mathrm{R}}}\right) N_{\mathrm{R}}^2 S_{t_{\mathrm{R}}}^2, \tag{14}$$

$$V_{\mathrm{M}} = \frac{N_{\mathrm{R}}}{n_{\mathrm{R}}} \sum_{r \in U_{\mathrm{R}}} N_{\mathrm{M}_r}^2 \left(\frac{1}{n_{\mathrm{M}_r}} - \frac{1}{N_{\mathrm{M}_r}}\right) S_{t_{M|r}}^2, \tag{15}$$

$$V_{\mathrm{F}} = \frac{N_{\mathrm{R}}}{n_{\mathrm{R}}} \sum_{r \in U_{\mathrm{R}}} \frac{N_{\mathrm{M}_r}}{n_{\mathrm{M}_r}} \sum_{m \in U_{\mathrm{M}_r}} \left[N_{F_{m_r}}^2 \left(\frac{1}{n_{F_{m_r}}} - \frac{1}{N_{F_{m_r}}}\right) n_{F_{mr}} S_{u_{|mr}}^2\right], \tag{16}$$

where

$$S_{t_{\mathrm{R}}}^2 = \frac{1}{N_{\mathrm{R}} - 1} \sum_{r \in U_{\mathrm{R}}} (t_r - \bar{t})^2; \quad t_r = \sum_{m \in U_{\mathrm{M}_r}} \sum_{f \in U_{F_{mr}}} u_{rmf}; \quad \bar{t} = \frac{1}{N_{\mathrm{R}}} \sum_{r \in U_{\mathrm{R}}} t_r, \tag{17}$$

$$S_{t_{M|r}}^2 = \frac{1}{N_{\mathrm{M}_r} - 1} \sum_{m \in U_{\mathrm{M}_r}} (t_{m|r} - \bar{t}_{|r})^2; \quad t_{m|r} = \sum_{f \in U_{F_{mr}}} u_{rmf}; \quad \bar{t}_{|r} = \frac{1}{N_{\mathrm{M}_r}} \sum_{m \in U_{\mathrm{M}_r}} t_{m|r} \tag{18}$$

and

$$S_{u_{|mr}}^2 = \frac{1}{N_{F_{mr}} - 1} \sum_{f \in U_{F_{mr}}} (u_{rmf} - \overline{u}_{|mr})^2; \quad \overline{u}_{|mr} = \frac{1}{N_{F_{mr}}} \sum_{f \in U_{F_{mr}}} u_{rmf}. \tag{19}$$

The second term of (11) represents the probabilistic variation that would have been obtained had we had complete data. This can be expressed as

$$
\begin{aligned}
Var\left[E\left(\widehat{T}\middle| u\right)\right] &= Var\left[E\left(\sum_{r=1}^{N_{\mathrm{R}}}\sum_{m=1}^{N_{\mathrm{M}_r}}\sum_{f=1}^{N_{F_{mr}}} I_{\mathrm{F}_{rmf}} w_{\mathrm{F}_{rmf}} u_{rmf}\middle| u\right)\right] \\
&= Var\left[\sum_{r=1}^{N_{\mathrm{R}}}\sum_{m=1}^{N_{\mathrm{M}_r}}\sum_{f=1}^{N_{F_{mr}}} u_{rmf}\right] = Var\left[T\right].
\end{aligned} \tag{20}
$$

3 Estimation of the variance of $\widehat{T}$

In practice, only the final sample of facilities is known, as so u_{rmf} is only known for the sample. This implies that the variance (13) cannot be calculated. However, we can estimate it using different approaches. The first approach, is to analytically find an unbiased estimator. A second approach is to use bootstrap re sampling, which return a consistent estimator.

3.1 Analytic estimation of $E\left[Var\left(\widehat{T}\middle| u\right)\right]$

Under the underlying design, an unbiased estimator of the term (12) can be obtained analytically. An unbiased estimator of this is given by

$$
\widehat{V}(\widehat{T}) = \widehat{V}_{\mathrm{R}}(\widehat{T}) + \widehat{V}_{\mathrm{M}}(\widehat{T}) + \widehat{V}_{\mathrm{F}}(\widehat{T}). \tag{21}
$$

$$
\widehat{V}_{\mathrm{R}} = \left(\frac{1}{n_{\mathrm{R}}} - \frac{1}{N_{\mathrm{R}}}\right) N_{\mathrm{R}}^2 \widehat{S}^2_{t_{\mathrm{R}}}, \tag{22}
$$

$$
\widehat{V}_{\mathrm{M}} = \frac{N_{\mathrm{R}}}{n_{\mathrm{R}}} \sum_{r\in s_{\mathrm{R}}} N^2_{\mathrm{M}_r}\left(\frac{1}{n_{\mathrm{M}_r}} - \frac{1}{N_{\mathrm{M}_r}}\right)\widehat{S}^2_{t_{M|r}}, \tag{23}
$$

$$
\widehat{V}_{\mathrm{F}} = \frac{N_{\mathrm{R}}}{n_{\mathrm{R}}} \sum_{r\in s_{\mathrm{R}}} \frac{N_{\mathrm{M}_r}}{n_{\mathrm{M}_r}} \sum_{m\in s_{\mathrm{M}_r}} \left[N^2_{F_{mr}}\left(\frac{1}{n_{F_{mr}}} - \frac{1}{N_{F_{mr}}}\right) n_{F_{mr}} \widehat{S}^2_{u_{|mr}}\right], \tag{24}
$$

where

$$
\widehat{S}^2_{t_{\mathrm{R}}} = \frac{1}{n_{\mathrm{R}}-1}\sum_{r\in s_{\mathrm{R}}}\left(\widehat{t}_r - \widetilde{\widehat{t}}\right)^2; \quad \widehat{t}_r = \sum_{m\in s_{\mathrm{M}_r}} \frac{N_{\mathrm{M}_r}}{n_{\mathrm{M}_r}} \sum_{f\in s_{F_{mr}}} \frac{N_{F_{mr}}}{n_{F_{mr}}} u_{rmf}; \quad \widetilde{\widehat{t}} = \frac{1}{n_{\mathrm{R}}}\sum_{r\in s_{\mathrm{R}}} \widehat{t}_r, \tag{25}
$$

$$
\widehat{S}^2_{t_{M|r}} = \frac{1}{n_{\mathrm{M}_r}-1}\sum_{m\in s_{\mathrm{M}_r}} (\widehat{t}_{m|r} - \widetilde{\widehat{t}}_{|r})^2; \quad \widehat{t}_{m|r} = \sum_{f\in s_{F_{mr}}} \frac{N_{F_{mr}}}{n_{F_{mr}}} u_{rmf}; \quad \widetilde{\widehat{t}}_{|r} = \frac{1}{n_{\mathrm{M}_r}} \sum_{m\in s_{\mathrm{M}_r}} \widehat{t}_{m|r}, \tag{26}
$$

and

$$
\widehat{S}^2_{u_{|mr}} = \frac{1}{n_{F_{mr}}-1}\sum_{f\in s_{F_{mr}}} (u_{rmf} - \widetilde{u}_{|m_r})^2; \quad \widetilde{u}_{|m_r} = \frac{1}{n_{F_{mr}}} \sum_{f\in s_{F_{mr}}} u_{rmf}. \tag{27}
$$

3.2 Analytic estimation of $Var\left[E\left(\widehat{T}\middle| u\right)\right]$

Under the underlying design, an unbiased estimator of the term (20) can be obtained analytically. An consistent estimator of this is given by

$$
\widehat{Var}\left[E\left(\widehat{T}\middle| u\right)\right] = \sum_{r=1}^{N_{\mathrm{R}}} I_{\mathrm{R}_r} \sum_{m=1}^{N_{\mathrm{M}_r}} I_{\mathrm{M}_{rm}} \sum_{f=1}^{N_{F_{mr}}} \frac{I_{\mathrm{F}_{rmf}}}{\pi_{\mathrm{F}_{rmf}}} u^2_{rmf} - N\left(\frac{1}{N}\sum_{r=1}^{N_{\mathrm{R}}} I_{\mathrm{R}_r} \sum_{m=1}^{N_{\mathrm{M}_r}} I_{\mathrm{M}_{rm}} \sum_{f=1}^{N_{F_{mr}}} \frac{I_{\mathrm{F}_{rmf}}}{\pi_{\mathrm{F}_{rmf}}} u_{rmf}\right)^2. \tag{28}
$$

Or an unbiased estimator is given by

$$
\widehat{Var}\left[E\left(\widehat{T}\middle| u\right)\right] = \sum_{r=1}^{N_{\mathrm{R}}} I_{\mathrm{R}_r} \sum_{m=1}^{N_{\mathrm{M}_r}} \frac{I_{\mathrm{M}_{rm}} N_{F_{mr}}}{\pi_{\mathrm{M}_{rm}}} \widehat{S}^2_{u_{|mr}} = \sum_{r\in s_{\mathrm{R}}}\sum_{m\in s_{\mathrm{M}_r}} \frac{N_{\mathrm{R}} N_{\mathrm{M}_r}}{n_{\mathrm{R}} n_{\mathrm{M}_r}} N_{F_{mr}} \widehat{S}^2_{u_{|mr}}. \tag{29}
$$

4 Post-design methods: Calibrated weights

The main purpose of post-designs methods is to improve the estimates by reducing the variance, i. e to produce more efficient estimators. Post-design methods include techniques such as post stratification, estimation of weights and calibration of weights. The latter is known to yield more efficient estimates by adjusting the weights using information readily-available at first phase. In Brazil, for example, the total number of doses administered is known and this can be use to adjust the weights.

Breslow et al. [2009a] and Breslow et al. [2009b] proposed using calibration to adjust the sampling weights in case–cohort designs. Let v_{F}, v_{M} and v_{R} denote readily available variables at first phase and at the different levels. The former is a variable available for all units at the final stage, the second is available for all second stage units and the latter is available for all first-stage units, e.g regions. The main goal is to adjust weights such that estimates for the totals $\widehat{T}_{v_{\mathrm{F}}}$, $\widehat{T}_{v_{\mathrm{M}}}$ and $\widehat{T}_{v_{\mathrm{R}}}$ equal the actual totals $T_{v_{\mathrm{F}}}$, $T_{v_{\mathrm{M}}}$) and $T_{v_{\mathrm{R}}}$), but forcing the new weights ($\widehat{w}$) to be as close as possible to the design weights. For example, consider IPW estimates $\widehat{T}_{v_{\mathrm{F}}}$ and $\widehat{T}_{v_{\mathrm{M}}}$ based on the same weights used to estimate T with $\widehat{T}$, i.e.

$$\widehat{T}_{v_{\mathrm{F}}} = \sum_{r=1}^{n_{\mathrm{R}}} \sum_{m=1}^{n_{\mathrm{M}_r}} \sum_{f=1}^{n_{F_{m_r}}} w_{\mathrm{F}_{rmf}} v_{\mathrm{F}rmf}; \quad \widehat{T}_{v_{\mathrm{M}}} = \sum_{r=1}^{n_{\mathrm{R}}} \sum_{m=1}^{n_{\mathrm{M}_r}} w_{\mathrm{M}_{rm}} v_{\mathrm{M}rm}; \quad \widehat{T}_{v_{\mathrm{R}}} = \sum_{r=1}^{n_{\mathrm{R}}} w_{\mathrm{R}_r} v_{\mathrm{R}r}.$$

Since we know $T_{v_{\mathrm{F}}}$, $T_{v_{\mathrm{M}}}$ and $T_{v_{\mathrm{R}}}$ we can modify the weights to force

$$\widehat{T}_{v_{\mathrm{F}}} = \sum_{r=1}^{n_{\mathrm{R}}} \sum_{m=1}^{n_{\mathrm{M}_r}} \sum_{f=1}^{n_{F_{m_r}}} \widehat{w}_{\mathrm{F}_{rmf}} v_{\mathrm{F}rmf} = T_{v_{\mathrm{F}}}; \quad \widehat{T}_{v_{\mathrm{M}}} = \sum_{r=1}^{n_{\mathrm{R}}} \sum_{m=1}^{n_{\mathrm{M}_r}} \widehat{w}_{\mathrm{M}_{rm}} v_{\mathrm{M}rm} = T_{v_{\mathrm{M}}}; \quad \widehat{T}_{v_{\mathrm{R}}} = \sum_{r=1}^{n_{\mathrm{R}}} \widehat{w}_{\mathrm{R}_r} v_{\mathrm{R}r} = T_{v_{\mathrm{R}}}. \tag{30}$$

Intuitively, the modified weights ($\widehat{w}_{\mathrm{F}_{rmf}}$, $\widehat{w}_{\mathrm{M}_{rm}}$) contain information about the entire population of facilities and we can use the new weights to find a new estimate of T.

$$\widehat{T}_{cal} = \widehat{T}_{\mathrm{F},cal} + \widehat{T}_{\mathrm{M},cal} + \widehat{T}_{R,cal} + T_{\mathrm{C}} = \sum_{r=1}^{n_{\mathrm{R}}} \sum_{m=1}^{n_{\mathrm{M}_r}} \sum_{f=1}^{n_{F_{m_r}}} \widehat{w}_{\mathrm{F}_{rmf}} y_{\mathrm{F}_{rmf}} + \sum_{r=1}^{n_{\mathrm{R}}} \sum_{m=1}^{n_{\mathrm{M}_r}} \widehat{w}_{\mathrm{M}_{rm}} y_{\mathrm{M}_{rm}} + \sum_{r=1}^{n_{\mathrm{R}}} \widehat{w}_{\mathrm{R}_r} y_{\mathrm{R}_r} + T_{\mathrm{C}}.$$

Mathematically, a distance function $d(w, \widehat{w})$ measuring the distance from original weights(w) to new weights($\widehat{w}$) is minimized subject to the constrains (30). There are several options for choosing this distance. Substantial gains in accuracy depend on how correlated the calibration variables are with the quantity of interest [Deville and Sarndal, 1992]. In order to find the asymptotic variance of $\widehat{T}_{cal}$, we follow [Deville and Sarndal, 1992]. Choosing one the distance functions suggested by them ensures that we can write

$$\begin{aligned} N_{\mathrm{F}}^{-1}\left(\widehat{T}_{\mathrm{F},cal} - \widehat{T}_{F,reg}\right) &= O_p\left(n_{\mathrm{F}}^{-1}\right); \\ N_{\mathrm{M}}^{-1}\left(\widehat{T}_{\mathrm{M},cal} - \widehat{T}_{M,reg}\right) &= O_p\left(n_{\mathrm{M}}^{-1}\right); \\ N_{\mathrm{R}}^{-1}\left(\widehat{T}_{\mathrm{R},cal} - \widehat{T}_{\mathrm{R},reg}\right) &= O_p\left(n_{\mathrm{R}}^{-1}\right), \end{aligned} \tag{31}$$

where $\widehat{T}_{F,reg}$, $\widehat{T}_{M,reg}$ and $\widehat{T}_{R,reg}$ are the so-called regression estimators shown in [Deville and Sarndal, 1992] given by

$$\begin{aligned} \widehat{T}_{F,reg} &= \sum_{r=1}^{n_{\mathrm{R}}} \sum_{m=1}^{n_{\mathrm{M}_r}} \sum_{f=1}^{n_{F_{m_r}}} \widetilde{w}_{\mathrm{F}_{rmf}} y_{\mathrm{F}_{rmf}}; \\ \widehat{T}_{M,reg} &= \sum_{r=1}^{n_{\mathrm{M}}} \sum_{m=1}^{n_{\mathrm{M}_r}} \widetilde{w}_{\mathrm{M}_{rm}} y_{\mathrm{M}_{rm}}; \\ \widehat{T}_{R,reg} &= \sum_{r=1}^{n_{\mathrm{R}}} \widetilde{w}_{\mathrm{R}_r} y_{\mathrm{R}_r} \end{aligned} \tag{32}$$

and

$$\begin{aligned}
&\widetilde{w}_{\mathrm{F}_{rmf}} = w_{\mathrm{F}_{rmf}}\left(1+v_{\mathrm{F}_{rmf}}^T\widetilde{\boldsymbol{\lambda}}_{\mathrm{R}}\right); \quad \widetilde{\boldsymbol{\lambda}}_{\mathrm{R}} = \widetilde{\boldsymbol{T}}_{\mathrm{R}}^{-1}\left(T_{v_{\mathrm{F}}} - \widehat{T}_{v_{\mathrm{F}}}\right); \quad \widetilde{\boldsymbol{T}}_{\mathrm{F}} = \sum_{r=1}^{n_{\mathrm{R}}}\sum_{m=1}^{n_{\mathrm{M}_r}}\sum_{f=1}^{n_{F_{m_r}}} \widetilde{w}_{\mathrm{F}_{rmf}} v_{\mathrm{F}_{rmf}} v_{\mathrm{F}_{rmf}}{}^T;\\
&\widetilde{w}_{\mathrm{M}_{rm}} = w_{\mathrm{F}_{rm}}\left(1+v_{\mathrm{M}_{rm}}^T\widetilde{\boldsymbol{\lambda}}_{\mathrm{M}}\right); \quad \widetilde{\boldsymbol{\lambda}}_{\mathrm{M}} = \widetilde{\boldsymbol{T}}_{\mathrm{M}}^{-1}\left(T_{v_{\mathrm{M}}} - \widehat{T}_{v_{\mathrm{M}}}\right); \quad \widetilde{\boldsymbol{T}}_{\mathrm{M}} = \sum_{r=1}^{n_{\mathrm{R}}}\sum_{m=1}^{n_{\mathrm{M}_r}} \widetilde{w}_{\mathrm{M}_{rm}} v_{\mathrm{M}_{rm}} v_{\mathrm{M}_{rm}}{}^T; \qquad (33)\\
&\widetilde{w}_{\mathrm{R}_r} = w_{\mathrm{R}_r}\left(1+v_{\mathrm{R}_r}^T\widetilde{\boldsymbol{\lambda}}_{\mathrm{R}}\right); \quad \widetilde{\boldsymbol{\lambda}}_{\mathrm{F}} = \widetilde{\boldsymbol{T}}_{\mathrm{F}}^{-1}\left(T_{v_{\mathrm{F}}} - \widehat{T}_{v_{\mathrm{F}}}\right); \quad \widetilde{\boldsymbol{T}}_{\mathrm{R}} = \sum_{r=1}^{n_{\mathrm{R}}} \widetilde{w}_{\mathrm{R}_r} v_{\mathrm{R}_r} v_{\mathrm{R}_r}{}^T.
\end{aligned}$$

Using (31) we can write:

$$\begin{aligned}
N_{\mathrm{F}}^{-1}\left(\widehat{T}_{\mathrm{F},cal}\right) &= O_p\left(n_{\mathrm{F}}^{-1}\right) + N_{\mathrm{F}}^{-1}\left(\widehat{T}_{F,reg}\right);\\
N_{\mathrm{M}}^{-1}\left(\widehat{T}_{\mathrm{M},cal}\right) &= O_p\left(n_{\mathrm{M}}^{-1}\right) + N_{\mathrm{M}}^{-1}\left(\widehat{T}_{M,reg}\right); \qquad (34)\\
N_{\mathrm{R}}^{-1}\left(\widehat{T}_{\mathrm{R},cal}\right) &= O_p\left(n_{\mathrm{R}}^{-1}\right) + N_{\mathrm{R}}^{-1}\left(\widehat{T}_{R,reg}\right)
\end{aligned}$$

and

$$N_{\mathrm{F}}^{-1}\left(\widehat{T}_{\mathrm{F},cal}\right) + N_{\mathrm{M}}^{-1}\left(\widehat{T}_{\mathrm{M},cal}\right) + N_{\mathrm{R}}^{-1}\left(\widehat{T}_{\mathrm{R},cal}\right) = O_p\left(n_{\mathrm{F}}^{-1}\right) + N_{\mathrm{F}}^{-1}\left(\widehat{T}_{F,reg}\right) + O_p\left(n_{\mathrm{M}}^{-1}\right) + N_{\mathrm{M}}^{-1}\left(\widehat{T}_{M,reg}\right) + O_p\left(n_{\mathrm{R}}^{-1}\right) + \text{[illegible]} \qquad (35)$$

4.0.1 Variance estimation

In order to estimate the variance in practice we use the expression

$$\widehat{T}_{cal} \approx \widehat{T}_{F,reg} + \widehat{T}_{M,reg} + \widehat{T}_{R,reg} + T_{\mathrm{C}} \qquad (36)$$

where, using Taylor expansion as in [Sarndal et al., 1992], we obtain that the approximate variance is th[illegible] same as the variance of

$$\sum_{r=1}^{n_{\mathrm{R}}}\sum_{m=1}^{n_{\mathrm{M}_r}}\sum_{f=1}^{n_{F_{m_r}}} w_{\mathrm{F}_{rmf}} E_{\mathrm{F}rmf} + \sum_{r=1}^{n_{\mathrm{R}}}\sum_{m=1}^{n_{\mathrm{M}_r}} w_{\mathrm{M}_{rm}} E_{\mathrm{M}rm} + \sum_{r=1}^{n_{\mathrm{R}}} w_{\mathrm{R}_r} E_{\mathrm{R}r}, \qquad (37)$$

with

$$\begin{aligned}
&E_{\mathrm{F}rmf} = y_{\mathrm{F}rmf} - v_{\mathrm{F}rmf}^T\boldsymbol{B}_{\mathrm{F}}; \quad \boldsymbol{B}_{\mathrm{F}} = \boldsymbol{T}_{\mathrm{F}}^{-1}\left(\sum_{r=1}^{N_{\mathrm{R}}}\sum_{m=1}^{N_{\mathrm{M}_r}}\sum_{f=1}^{N_{F_{m_r}}} v_{\mathrm{R}_{rmf}}\right)\\
&E_{\mathrm{M}rm} = y_{\mathrm{M}rm} - v_{\mathrm{F}rmf}^T\boldsymbol{B}_{\mathrm{F}}; \quad \boldsymbol{B}_{\mathrm{M}} = \boldsymbol{T}_{\mathrm{M}}^{-1}\left(\sum_{r=1}^{N_{\mathrm{R}}}\sum_{m=1}^{N_{\mathrm{M}_r}} v_{\mathrm{M}_{rm}}\right) \qquad (38)\\
&E_{\mathrm{R}r} = y_{\mathrm{R}r} - v_{\mathrm{R}r}^T\boldsymbol{B}_{\mathrm{R}}; \quad \boldsymbol{B}_{\mathrm{R}} = \boldsymbol{T}_{\mathrm{R}}^{-1}\left(\sum_{r=1}^{N_{\mathrm{R}}}\sum_{m=1}^{N_{\mathrm{M}_r}} v_{\mathrm{R}_{rm}}\right).
\end{aligned}$$

We now define

$$\widetilde{u}_{rmf} = e_{\mathrm{F}_{rmf}} + \frac{w_{\mathrm{M}_{rm}}}{w_{\mathrm{F}_{rmf}} n_{F_{mr}}} e_{\mathrm{M}_{rm}} + \frac{w_{\mathrm{R}_r}}{w_{\mathrm{F}_{rmf}} n_{\mathrm{F}_r}} e_{\mathrm{R}_r},$$

where $e_{\mathrm{F}_{rmf}}$, $e_{\mathrm{M}_{rm}}$ and e_{R_r} are estimates for $E_{\mathrm{F}_{rmf}}$, $E_{\mathrm{M}_{rm}}$ and E_{R_r}. They are given by

$$\begin{aligned}
&e_{\mathrm{F}rmf} = y_{\mathrm{F}rmf} - v_{\mathrm{F}rmf}^T\widetilde{\boldsymbol{B}}_{\mathrm{F}}; \quad \widetilde{\boldsymbol{B}}_{\mathrm{F}} = \widetilde{\boldsymbol{T}}_{\mathrm{F}}^{-1}\left(\sum_{r=1}^{N_{\mathrm{R}}}\sum_{m=1}^{N_{\mathrm{M}_r}}\sum_{f=1}^{N_{F_{m_r}}} v_{\mathrm{R}_{rmf}}\right)\\
&e_{\mathrm{M}rm} = y_{\mathrm{M}rm} - v_{\mathrm{F}rmf}^T\widetilde{\boldsymbol{B}}_{\mathrm{F}}; \quad \widetilde{\boldsymbol{B}}_{\mathrm{M}} = \widetilde{\boldsymbol{T}}_{\mathrm{M}}^{-1}\left(\sum_{r=1}^{N_{\mathrm{R}}}\sum_{m=1}^{N_{\mathrm{M}_r}} v_{\mathrm{M}_{rm}}\right) \qquad (39)\\
&e_{\mathrm{R}r} = y_{\mathrm{R}r} - v_{\mathrm{R}r}^T\widetilde{\boldsymbol{B}}_{\mathrm{R}}; \quad \widetilde{\boldsymbol{B}}_{\mathrm{R}} = \widetilde{\boldsymbol{T}}_{\mathrm{R}}^{-1}\left(\sum_{r=1}^{N_{\mathrm{R}}}\sum_{m=1}^{N_{\mathrm{M}_r}} v_{\mathrm{R}_{rm}}\right).
\end{aligned}$$

Therefore

$$\widehat{Var}\left(\widehat{T}_{cal}\right) = Var\left(\sum_{r=1}^{N_{\mathrm{R}}} I_{\mathrm{R}_r} \sum_{m=1}^{N_{\mathrm{M}_r}} I_{\mathrm{M}_{rm}} \sum_{f=1}^{N_{F_{mr}}} I_{\mathrm{F}_{rmf}} w_{\mathrm{F}_{rmf}} \widetilde{u}_{rmf}\right) = \widehat{E}\left[Var\left(\widehat{T}_e \middle| u\right)\right] + \widehat{Var}\left[E\left(\widehat{T}_e \middle| u\right)\right], \quad (40)$$

with $\widehat{T}_e = \sum_{r=1}^{N_{\mathrm{R}}} I_{\mathrm{R}_r} \sum_{m=1}^{N_{\mathrm{M}_r}} I_{\mathrm{M}_{rm}} \sum_{f=1}^{N_{F_{mr}}} I_{\mathrm{F}_{rmf}} w_{\mathrm{F}_{rmf}} \widetilde{u}_{rmf}$. Expression (40) is found in a similar way as the variance in section 2.

REFERENCES

N. Breslow, T. Lumley, C. Ballantyne, L. Chambless, and M Kulich. Improved horvitz-thompson estimation of model parameters from two-phase stratified samples: Applications in epidemiology. *Statistics in Biosciences*, 1(1):32–49, 2009a.

Norman E. Breslow, Thomas S. Lumley, Christie M. Ballantyne, Lloyd Ellwood Chambless, and Michal Kulich. Using the whole cohort in the analysis of case-cohort data. *American journal of epidemiology*, 169(11):1398–405, 2009b.

J.C. Deville and C. E. Sarndal. Calibration estimators in survey sampling. *Journal of the American Statistical Association*, 87:376–382, 1992.

C. E. Sarndal, B. Swensson, and J. Wretman. *Model Assisted Survey Sampling*. New York, 1992.
